# Psychosocial and physiological health outcomes of outdoor green exercise versus indoor exercise in knee osteoarthritis patients coexisting with type 2 diabetes mellitus: a randomized controlled trial

**DOI:** 10.3389/fendo.2025.1560536

**Published:** 2025-05-14

**Authors:** Yilin Jin, Xiang Geng, Qiaojie Wang, Feng Xue, Shengdi Lu, Lihua Huang

**Affiliations:** ^1^ Department of Orthopedic Surgery, Huashan Hospital, Fudan University, Shanghai, China; ^2^ School of Nursing, Fudan University, Shanghai, China; ^3^ Department of Orthopedics, Shanghai Sixth People’s Hospital Affiliated to Shanghai Jiao Tong University School of Medicine, Shanghai, China; ^4^ Department of Rehabilitation, Shanghai Sixth People’s Hospital Affiliated to Shanghai Jiao Tong University School of Medicine, Shanghai, China

**Keywords:** green exercise, knee osteoarthritis, type 2 diabetes mellitus, psychosocial and physiological health, physical activity

## Abstract

**Background:**

Both physical activity and nature exposure are associated with several health benefits for both knee osteoarthritis (KOA) and type 2 diabetes mellitus (T2DM). However, the health outcomes when being physically active in nature, called green exercise (GE), are less clear. The purpose of this study is to evaluate the psychosocial and physiological outcomes for green exercise in KOA patients coexisting with T2DM compared to indoor exercise.

**Method:**

A prospective, randomized, open-label, parallel, multi-center clinical trial conducted at two hospitals in Shanghai that included 82 patients T2DM and KOA. Enrollment occurred between January 2020 and March 2022, and follow-up was completed October 2022. Participants were randomized to outdoor green cycling (OGC) group and indoor stationary cycling (ISC) group. Psychosocial and physiological health outcomes were evaluated through questionnaires comprising standard international measures of self-esteem (Rosenberg Self-esteem Scale [RSE]), perceived stress (Perceived Stress Scale [PSS]), mood (Profile of Mood States [POMS]) and nature relatedness (Nature Relatedness Scale – short-form version). Participants’ enjoyment of exercise and intention for a future exercise behavior was measured in a questionnaire using a 100mm visual analogue scale as a continuum from 0 – ‘not at all’ to 100 – ‘very much’.

**Results:**

Among 82 patients, 74 (90.2%) completed the trial. By 24 weeks, the OGC group showed a greater reduction in perceived stress (3.7 vs. 2.8, P = 0.007), and total mood disturbance (POMS) scores decreased more in the OGC group (-7.2 vs. -6.1, P = 0.030). Tension and depression subscales also showed greater reductions in the OGC group (P < 0.001 for both), along with higher improvements in vigor (10.2 vs. 8.1, P < 0.001). Enjoyment of exercise was slightly higher in the OGC group, though this difference did not reach statistical significance (P = 0.061), and their intention to continue exercising was also significantly stronger (P = 0.021). Participants in OGC group achieved significant decrease of HbA1c than participants in ISC group.

**Conclusion:**

Green exercise offers an accessible provision for improving short-term psychological wellbeing than indoor exercises.

**Clinical Trial Registration:**

https://www.chictr.org.cn/, identifier ChiCTR2100042872.

## Introduction

Multimorbidity presents a significant public health challenge with crucial implications for health management and policy. A prominent pattern of multimorbidity is the coexistence of cardiometabolic and osteoarticular diseases, notably the common combination of type 2 diabetes mellitus (T2DM) and osteoarthritis (OA) (1). The association between T2DM and knee osteoarthritis (KOA) has received attention due to their concurrent prevalence and mutual risk factors, such as obesity and aging. Studies show a robust link between T2DM and KOA, with one study finding that people with T2DM are more than twice as likely to develop KOA compared to those without diabetes, especially among non-obese individuals (2). This suggests that the impact of diabetes on KOA extends beyond the influence of obesity alone. The body of research underscores a substantial connection between T2DM and KOA, indicating that the underlying mechanisms may transcend common risk factors like obesity (3, 4).

Exercise is recognized as a fundamental component of managing T2DM, alongside diet and effective medications (5, 6). The benefits of exercise, including improved glycemic control and better blood lipid profiles, are well-supported by evidence (7–9). However, the relative benefits of different exercise types remain less certain. Aerobic exercise, which involves activities like brisk walking, cycling, swimming, and jogging that use large muscle groups, is the most extensively studied form of exercise for this purpose (8).

Particularly, the type of exercise environment is believed to play a crucial role in the emotional and stress-reducing effects of short-term exercise sessions. Exercise performed in natural, “green” settings leads to notably better outcomes in mood, focus, and physiological responses compared to exercising indoors or in outdoor “built” environments, where buildings and synthetic materials are predominant (10–15). During physical activity, natural settings are most effective in rejuvenating fatigued cognitive directed attention (the deliberate mental capacity to resist distractions from competing stimuli) (16, 17). This rejuvenation is advantageous for workplace task performance, and it may explain why short episodes of exercise in nature enhance mood and emotional states (18–20).

Psychosocial factors, such as depression, have been linked to the development of T2DM and to poorer results on glucose tolerance tests (21). Additionally, the presence of depression at the start of a study may predict the emergence of impaired glucose regulation and T2DM (22). On the other hand, a systematic review and meta-analysis recently reported that 19.9% of people with OA had depressive symptoms, with a relative risk of depression of 1.17 in those with OA compared to those without (23, 24). However, depression is often under-recognized and under-treated in older adults, particular in patients with OA (25, 26).

To our knowledge, no research has examined the psychosocial and physiological outcomes of outdoor green exercise for patients with multimorbidity of both KOA and T2DM. The purpose of this study was to compare different exercise environments for patients with KOA and T2DM in terms of psychosocial and physiological change, glycemic control, and functional outcomes. The hypotheses were that: 1) The enhancement in mood from pre- to post-exercise will be more apparent in outdoor green exercise settings than in indoor ones; 2) Participants engaging in outdoor green exercise will exhibit superior physiological status during their workout sessions compared to those exercising indoors; 3) Outdoor green exercise environments will lead to a more significant improvement in patients’ blood glucose levels compared to indoor exercise settings; 4) Following outdoor green exercise, participants will demonstrate better functional mobility than those who undertake indoor exercise.

## Materials and methods

### Study design

This is a prospective, randomized, open-label, parallel, multi-center clinical trial. Eligible participants were recruited from Shanghai Sixth People’s Hospital Affiliated to Shanghai Jiao Tong University School of Medicine and Huashan Hospital Affiliated to Fudan University. All participants were divided into OGC group and ISC group. The allocation ratio was 1:1. This trial was registered at Chinese Clinical Trial before the first recruitment of the participants (ID: ChiCTR2100042872). This trial was approved by the Institutional Review Board of Shanghai Sixth People’s Hospital Affiliated to Shanghai Jiao Tong University School of Medicine (IRB No: 2019-KY-063(K)) and other trial sites acknowledged the IRB approval. All participants provided written informed consents.

### Study participants

KOA was confirmed according to the criteria from NICE (27): Osteoarthritis should be diagnosed clinically without the need for imaging in individuals over 45 years who exhibit activity-related joint pain and either no morning stiffness or stiffness lasting less than 30 minutes.

The definition of T2DM in the present study was formulated according to the SUPREME-DM (28) criteria as follows: a) 1 or more of the International Classification of Disease, Ninth Revision, Clinical Modification (ICD-9-CM) codes and Tenth Revision, Clinical Modification (ICD-10-CM) codes for type 2 diabetes associated with in-patient encounters; b) 2 or more ICD codes associated with out-patient encounters on different days within 2 years; c) combination of 2 or more of the following associated with out-patient encounters on different days within 2 years: 1) ICD codes associated with out-patient encounters; 2) fasting glucose level ≥ 126 mg/dl; 3) 2-hour glucose level ≥ 200 mg/dl; 4) random glucose ≥ 200 mg/dl; 5) HbA1c ≥ 6.5%; and 6) prescription for an antidiabetic medication.

### Interventions

Before the baseline assessment, all participants received standard care at the Knee Pain Specialized Clinic of Shanghai Sixth People’s Hospital affiliated to Shanghai Jiao Tong University School of Medicine or Huashan Hospital Affiliated to Fudan University. The usual care for KOA included diagnosis, providing information about the condition, identifying personal risk factors, and developing a tailored treatment plan. This plan typically involved pain medication and advice on knee protection for daily life activities.

To ensure the effectiveness of the cycling exercise and to reduce the risk of injury, all participants were required to set their posture correctly under the guidance of rehabilitation staff before beginning the exercise (see **Supplementary Material**).

#### Indoor stationary cycling group

Participants in the ISC group were instructed to use stationary cycling for three times per week for 30–40 minutes. Investigators individualized the program for each participant by estimating target exercise heart rate for the sessions and providing heart rate monitors to help subjects adhere to exercise guidelines (see **Supplementary Material**).

#### Outdoor green cycling group

Participants in the OGC group were required to exercise for three times per week for 30–40 minutes step-by-step in groups of maximally 4 people supervised by a physical therapist. Each participant wears a heart rate monitor band around the chest to ensure reaching the target exercise heart rate (see **Supplementary Material**).

### Outcomes

Psychosocial and physiological health outcomes included pre-post changes of Rosenberg Self-Esteem Scale (RSE), Perceived Stress Scale (PSS), and Profile of Mood States (POMS). The RSE is one of the most widely used instruments for measuring self-esteem, which consists of 10 items, rated on a four-point Likert scale ranging from “Strongly Agree” to “Strongly Disagree.” Five of the items are positively worded (e.g., “I feel that I have a number of good qualities”) and five are negatively worded (e.g., “I feel I do not have much to be proud of”). This balance is intended to control for acquiescence bias, where respondents may agree with statements regardless of their content (29).

The PSS is a psychological instrument designed to measure the perception of stress. Developed by Sheldon Cohen and his colleagues in 1983, the PSS assesses the degree to which situations in one’s life are appraised as stressful. It is widely used in psychological research to evaluate the role of stress in the etiology of disease and behavioral disorders. The PSS consists of 10 items (PSS-10), though original versions included 14 items (PSS-14) and a shorter version of 4 items (PSS-4) is also available. Respondents are asked to rate their feelings and thoughts during the last month on a 5-point Likert scale ranging from “Never” to “Very Often” (30).

The POMS is a psychological rating scale used to assess transient, distinct mood states. Developed by Douglas M. McNair, Maurice Lorr, and Leo F. Droppleman in 1971, the POMS is widely utilized in clinical psychology, sports psychology, and research to measure mood fluctuations over a specific period. The POMS originally consists of 65 items, each describing a mood-related adjective (e.g., “angry,” “energetic”). Respondents rate each item on a 5-point Likert scale ranging from “Not at All” to “Extremely,” based on how they have been feeling during the past week, including the day of assessment (31).

Primary functional outcome was the pre-post changes of HbA1c. The secondary functional outcome was changes of Knee Injury and Osteoarthritis Outcome Score (KOOS) from baseline. KOOS is a patient-reported outcome measurement system used to evaluate short-term and long-term symptoms and function in individuals with knee injuries and osteoarthritis. The score consisted of 5 separately scored subscales; Pain, Symptoms, Function in daily living (ADL), Function in Sport and Recreation (Sport/Rec) and knee-related Quality of Life (QoL). The score ranges from 0 to 100 with 0 representing extreme problems and 100 representing no problems. Other secondary outcome included changes in body mass index (BMI).

### Sample size

For a two-arm trial with the primary outcome measured by RSE Scale. At the significance level of 0.05, with a noninferiority margin of 5, 36 subjects for each group are needed to achieve 80% power when the mean response difference between treatment and control is 1 and the standard deviation is assumed to be 10 (32). The sample size was *a priori* increased to at least 40 participants for each group adjusted for a 10% dropout rate (33).

### Randomization, blinding and follow-up

The participants were randomly assigned to the intervention and control groups in a 1:1 ratio after screening. The randomization sequence was generated by the institutional staff and was concealed from the physical therapists during follow‐up. Psychosocial and physiological health and functional data were collected from the participants at baseline and at the six weeks and sixth months after initiation of exercise; these data included sex, use of walking aid, BMI, and history of diseases and medications. Height and weight were measured using standardized methods. BMI was calculated as the weight in kilograms divided by the squared height in meters. Other data were collected from questionnaires.

No blinding was performed in this trial. Only the analyst who assessed the outcomes was blind to this trial.

### Statistical analysis

The analysis was performed according to the per-protocol principle and the adverse events intention-to-treat (ITT) population. The normality of the data was tested using the Shapiro-Wilk test. Measures that followed a normal distribution were presented as means and standard deviations, and comparisons between groups were conducted using the two independent samples t-test. The effect size was calculated as Cohen’s d for primary outcomes. Variables that did not follow a normal distribution were presented as medians and interquartile ranges, and comparisons between the groups were conducted using the Wilcoxon rank-sum test. Categorical data were presented as frequencies (number of cases) or percentages, and group comparisons were performed using the chi-square test or Fisher’s exact test, depending on the number of categories (columns) and the sample size in the R×C contingency table. The above data were analysed using R (version 4.3.3) and a two-sided test (α=0.05).

## Results

### Baseline characteristics of the study participants

**Figure 1:**
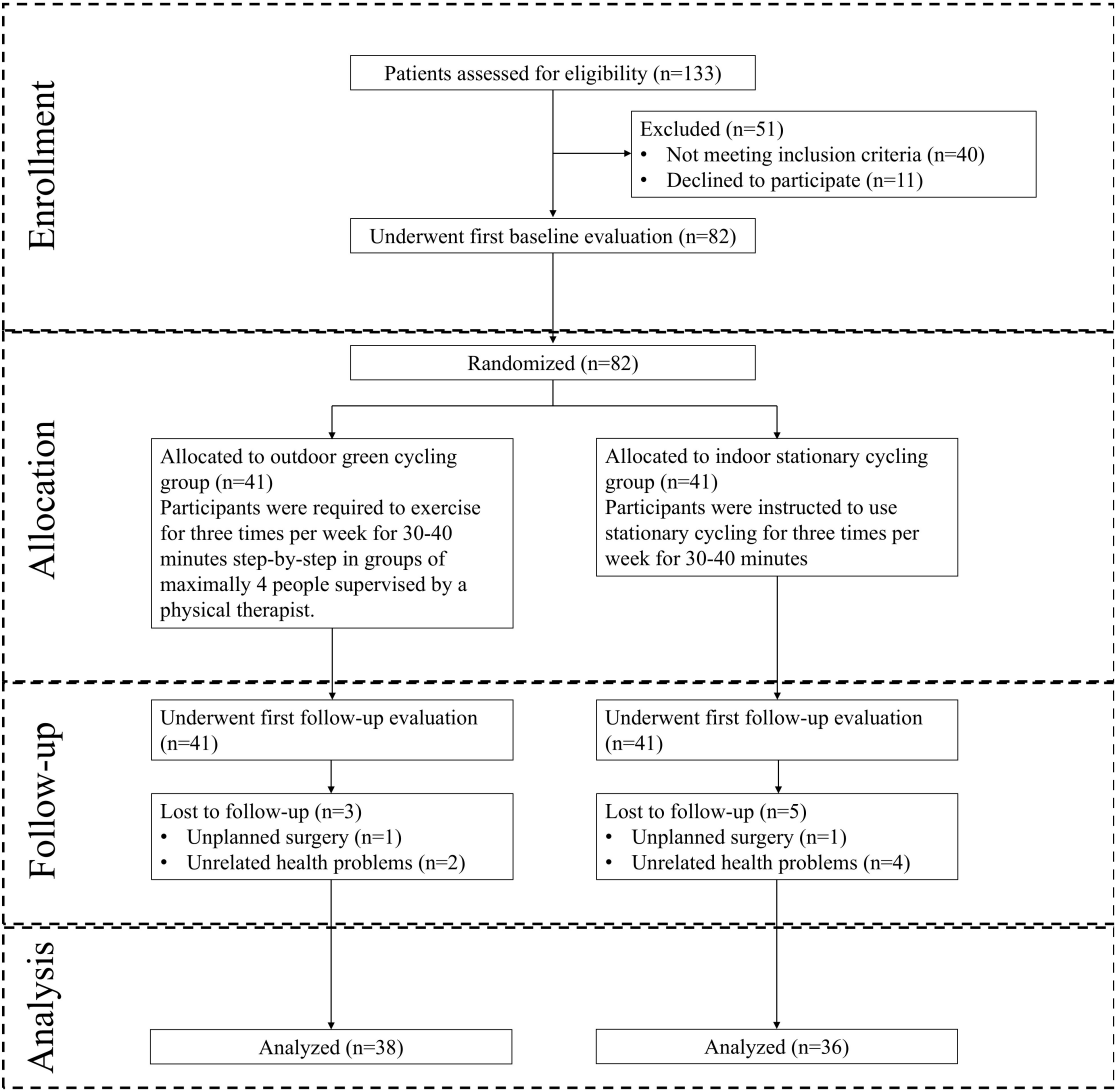
Flowchart of the study.

Of the 133 participants who underwent screening in this study, 82 were enrolled into the final analysis and randomized. 41 were in the ISC group, 41 were in the OGC group. Of these, a total of 74 (90.2%) completed the full follow-up visits. 8 participants (5 in ISC group, 3 in OGC group) failed to complete the entire study due to unplanned surgery and injury (N = 2) and reasons unrelated to the study (N = 6) (**Figure 1**).

The baseline characteristics are shown in **Table 1**. The demographic and clinical characteristics of the study participants at baseline show that the OGC group (N = 38) and the ISC group (N = 36) were well-matched. The mean age was 57.6 years in the OGC group and 58.1 years in the ISC group (P = 0.740), and both groups had a similar percentage of males (55.3% vs. 55.6%, P = 0.980). The average BMI was also similar between the groups (24.8 kg/m² in the OGC group and 24.6 kg/m² in the ISC group, P = 0.755). Insurance type and comorbid illnesses such as hypertension and cardiovascular disease were comparable across both groups, with no significant differences (**Table 1**).

**Table 1:** Demographic and clinical characteristics of the study participants at baseline.

Variables	Outdoor green cycling group (N=38)	Indoor stationary cycling group (N=36)	P value
Age (years), mean ± SD	57.6 ± 6.2	58.1 ± 6.7	0.740
Gender, Male, N. (%)	21 (55.3)	20 (55.6)	0.980
BMI (kg/m^2^), mean ± SD	24.8 ± 2.8	24.6 ± 2.7	0.755
Education level, N. (%)			
High School or University	24 (63.2)	25 (69.4)	0.568
Insurance type, N. (%)			0.753
Government	33 (86.8)	32 (88.9)	
Commercial	1 (2.6)	2 (5.6)	
Self-financed	4 (10.5)	2 (5.6)	
HbA1c level, mean ± SD	9.1 ± 1.1	8.9 ± 1.2	0.458
Comorbid illness*, N. (%)			0.965
Hypertension	26 (68.4)	24 (66.7)	
Cardiovascular disease	12 (31.6)	10 (27.8)	
Arthritis in other joints	6 (15.8)	6 (16.7)	
Kellgren-Lawrence grade**, N. (%)			0.656
2	21 (55.3)	23 (63.9)	
3	15 (39.5)	12 (33.3)	
4	2 (5.3)	1 (2.8)	
Patellofemoral OA, severity 1,2 (mild-moderate)***, N. (%)	28 (73.7)	28 (77.8)	0.682
Paracetamol and NSAID, N. (%)	20 (52.6)	21 (58.3)	0.622
Walking aid, N. (%)	2 (5.3)	1 (2.8)	1.000

Data were presented as mean ± SD for continuous variables, or number (percentage) for categorical variables.

SD, standard deviation; BMI, body mass index; OA, osteoarthritis; NSAID, non-steroid anti-inflammatory drug.

*Reported on a self-administered health history questionnaire (with the exception of patellofemoral OA) as conditions diagnosed by a health care professional. With comorbid illnesses that could exclude patients from participation, final approval or denial for participation provided after patient evaluation by study physician.

**The Kellgren-Lawrence scale ranges from 0 to 4. A grade of 2 or greater indicates definite osteoarthritis on posteroanterior weight-bearing radiograph. A grade of 2 indicates definite osteophytes and possible joint space narrowing; grade 3, multiple osteophytes, definite joint space narrowing, sclerosis, and possible bony deformity; and grade 4, large osteophytes, marked definite joint space narrowing, severe sclerosis, and definite bony deformity.

***Patellofemoral OA measured from skyline view radiograph using the OARSI scale (0, none; 1, mild; 2, moderate; 3, severe). Patients with severe (scale=3) patellofemoral OA were excluded. One patient was missing baseline skyline view radiographs.

### The changes in RSS, PSS and POMS

Significant improvements were seen in the OGC group. By week 24, the OGC group experienced a greater reduction in perceived stress compared to the ISC group (3.7 vs. 2.8, P = 0.007). Additionally, the total mood disturbance (POMS) score showed a larger decrease in the OGC group (-7.2 vs. -6.1, P = 0.030). Tension and depression subscales also showed greater reductions in the OGC group (P < 0.001 for both), along with higher improvements in vigor (10.2 vs. 8.1, P < 0.001). Enjoyment of exercise was marginally higher in the OGC group (P = 0.061), and their intention to continue exercising was also significantly stronger (P = 0.021) (**Table 2**).

**Table 2:** Changes in psychosocial and physiological health outcomes at 6 weeks and 24 weeks from baseline in per-protocol population.

Outcome	Outdoor green cycling group (N=38)	Indoor stationary cycling group (N=36)	P value	Effect Size**
Self-esteem (RSE) change				
From baseline to 6-wk	2.6 ± 1.1	2.2 ± 0.9	0.508	0.40
From baseline to 24-wk	3.3 ± 1.3	2.6 ± 1.2	0.011	0.56
Perceived Stress Scale (PSS) change				
From baseline to 6-wk	3.2 ± 1.4	2.5 ± 1.2	0.031	
From baseline to 24-wk	3.7 ± 1.6	2.8 ± 1.4	0.007	
Total MOOD Disturbance (POMS) change				
From baseline to 6-wk	-5.3 ± 1.7	-5.0 ± 1.6	0.383	
From baseline to 24-wk	-7.2 ± 2.1	-6.1 ± 1.8	0.030	
POMS subscales change (from baseline to 24-wk)				
Tension	-28.5 ± 6.8	-22.7 ± 5.6	<0.001	
Depression	-4.5 ± 0.9	-3.7 ± 0.8	<0.001	
Anger	-5.1 ± 1.1	-4.7 ± 1.0	0.075	
Vigor	10.2 ± 2.5	8.1 ± 1.9	<0.001	
Fatigue	-6.6 ± 2.3	-5.9 ± 2.4	0.175	
Confusion	-3.1 ± 1.7	-2.0 ± 0.8	0.001	
*Enjoyment of current exercise	65.9 ± 23.5	63.1 ± 22.4	0.061	
*Intention for a future exercise	69.6 ± 22.1	57.7 ± 21.1	0.021	

Data were presented as mean ± SD for continuous variables.

RSE, Rosenberg Self-esteem Scale; PSS, Perceived Stress Scale; POMS, Profile of Mood States.

*A 100 mm line was employed to assess participants’ enjoyment of the exercise and their intention to engage in future exercise behavior. For enjoyment, participants were asked to place a mark on the line reflecting their level of enjoyment of the session, with the scale ranging from “not at all” (0) to “very much” (100). Similarly, to gauge intention, participants answered the question, “Would you attend a free exercise session in the same location where you did your cycling today?” by marking their intention on the line. The scale for this measure spanned from “I definitely will not attend” (0) to “I will definitely attend” (100). Participants marked their responses on the 100-mm continuum lines for each question, which were included only in the post-exercise questionnaire.

**Effect Size Calculation (Cohen’s *d*): per Cohen’s convention: *d* = 0.2 = small, 0.5 = medium, 0.8 = large.

### The changes in HbA1c

Pre-post changes of HbA1c were significant in 6- and 24-weeks follow-up in two groups respectively. Participants in OGC group achieved significant decrease of HbA1c than participants in ISC group (**Table 3**).

**Table 3:** Changes in functional and other outcome measures at 6 weeks and 24 weeks from baseline in per-protocol population.

Outcome	Outdoor green cycling group (N=38)	Indoor stationary cycling group (N=36)	P value
HbA1c level (%)			
From baseline to 6-wk	-0.9 ± 0.2	-0.7 ± 0.2	< 0.001
From baseline to 24-wk	-1.6 ± 0.3	-1.3 ± 0.3	< 0.001
BMI (kg/m^2^)			
From baseline to 6-wk	1.8 ± 0.4	1.6 ± 0.4	0.015
From baseline to 24-wk	2.8 ± 0.8	2.3 ± 0.6	0.006
KOOS pain			
From baseline to 6-wk	2.1 ± 0.5	2.0 ± 0.5	0.502
From baseline to 24-wk	3.3 ± 0.7	3.1 ± 0.6	0.321
KOOS symptoms			
From baseline to 6-wk	4.9 ± 1.2	4.8 ± 1.0	0.787
From baseline to 24-wk	7.8 ± 2.2	7.4 ± 2.0	0.343
KOOS ADL			
From baseline to 6-wk	7.4 ± 2.3	6.9 ± 2.9	0.375
From baseline to 24-wk	13.3 ± 3.5	10.9 ± 2.8	0.001
KOOS Sport/Rec			
From baseline to 6-wk	3.1 ± 0.9	2.9 ± 0.8	0.320
From baseline to 24-wk	5.5 ± 1.7	3.7 ± 1.2	< 0.001
KOOS QoL			
From baseline to 6-wk	3.8 ± 1.2	3.3 ± 1.1	0.069
From baseline to 24-wk	6.4 ± 2.1	4.8 ± 1.3	< 0.001

BMI, Body Mass Index; KOOS, Knee Injury and Osteoarthritis Outcome Score; ADL, activities of daily living; Sport/Rec, Sport and Recreation; QoL, Quality of Life.

### The changes in KOOS

BMI reduction was more pronounced in the OGC group at both time points (2.8 kg/m² vs. 2.3 kg/m² at 24 weeks, P = 0.006). Improvements in KOOS scores for quality of life (6.4 vs. 4.8, P < 0.001), and Sport/Rec (5.5 vs. 3.7, P < 0.001) were significantly better in the OGC group by 24 weeks (**Table 3**).

### Adherence and Intention

The result found that enjoyment of current exercise was similar between two groups, but intention for future exercise behavior was significantly positively associated with participants who engaged in outdoor green exercise (**Table 2**).

### Adverse events

During the follow-up period, a similar proportion of participants in both groups reported adverse events. No serious events were related to intervention, while two minor events were possibly related to the outdoor green cycling intervention (**Table 4**). The proportions of participants lost to follow-up were equivalent in both groups, with most losses occurring at the final follow-up survey (**Figure 1**).

**Table 4:** Adverse events and serious adverse events.

Adverse events	Outdoor green cycling group (N=41)	Indoor stationary cycling group (N=41)
Patients with adverse events (no. [%])	6 (14.6)	8 (19.5)
Events related to study therapy (no.)	2#	1
Events unrelated to study therapy (no.)	6	7
Type of event (no.)		
Involved knee		
Pain	2	3
Bruising	2	1
Swelling	0	2
Other		
Fall with minor symptoms	2	0
Nausea and dizziness	0	0
Back pain	1	0
Anxiety about knee recovery	1	1
Serious adverse events*		
Patients with serious adverse events (no. [%])	3 (7.3)	5 (12.2)
Events related to study therapy (no.)	0	0
Events unrelated to study therapy (no.)	3	5
Type of event (no.)		
Hospitalization	1	2
Degradation of the general condition	1	1**
Hip fracture due to fall	1**	0
Waist fracture due to fall	1	1
Cardiac arrhythmia	0	2
Spinal surgery	0	1**

#Two patients fell during intervention with minor consequent symptoms (pain and bruise).

*Patients with serious adverse events were automatically withdrawn from the study.

**Events related to hospitalization.

## Discussion

Our randomized, controlled trial involving T2DM patients showed that in 24 weeks, the OGC group showed significantly greater reductions in perceived stress, mood disturbance, and depression compared to the ISC group (P < 0.05). Additionally, the Green outdoor group experienced higher vigor and tension reduction, indicating better psychological benefits​. Our hypothesis that greater intention for a future exercise behavior and greater enjoyment of the exercise session would be reported following outdoors green exercise compared to indoors exercise were confirmed based on the results of this study. To our knowledge, this is the first study to examine the differential psychosocial and physiological effects of GE compared to indoor training on patients with chronic diseases.

Both OA and T2DM are complex diseases influenced by genetic, demographic, and lifestyle factors, such as older age and obesity (34). Older age, in particular, significantly impacts the prevalence and severity of both OA and T2DM. As individuals age, the cumulative wear and tear on joint cartilage increases the likelihood of OA development, resulting in joint pain and stiffness. Similarly, the prevalence of T2DM rises with age due to progressive beta-cell dysfunction and increasing insulin resistance over time (29, 30, 35, 36). Lifestyle intervention is considered a cornerstone of treatment of both OA and T2DM (4, 8, 31, 37). Effective weight management through dietary modifications and regular physical activity is essential to reduce joint stress and enhance mobility in OA patients. Targeted exercise programs that improve muscle strength and joint flexibility can significantly alleviate pain and enhance functional outcomes. For T2DM, lifestyle interventions focusing on a balanced diet and consistent physical activity are critical for managing blood glucose levels and improving overall metabolic health (38, 39).

On the other hand, physical activity has been shown to release endorphins and other neurochemicals that promote feelings of well-being and reduce symptoms of depression and anxiety (31, 37–39). These benefits are especially pronounced in natural environments (40), which can be especially advantageous for patients managing chronic conditions such as T2DM and OA. In our study, participants in both groups reported reduced levels of depression and anxiety following the initiation of exercise; however, participants in the GE group experienced significantly greater improvements, with statistically significant differences observed. Our results align with previous research. For example, Rogerson et al. demonstrated that a single session of GE improved self-esteem by 7.7%, reduced stress by 18.4%, and enhanced mood by 14.2% (41). Additionally, the observed improvements in tension, depression, vigor, and confusion are consistent with prior findings (42). While these measures may not fully reflect the long-term benefits or clinical significance for participants, they do indicate meaningful improvements in acute psychological well-being. In the short term, such results can provide valuable insights for health promotion initiatives (43). Previous studies have also found that immediate emotional responses to exercise play a critical role in fostering long-term motivation and adherence to regular physical (44, 45).

Here, participation in GE might serve as a tool for maintaining exercise behaviors (10). The increase in POMS fatigue contradicts previous studies. The 40-minute cycling session in our study is likely more intense than the exercises in earlier GE research (11, 12). Higher exercise intensity may lead participants to interpret items on the fatigue subscale (fatigued, worn out, exhausted, sluggish, weary) in a physiological sense rather than the intended psychological sense. Although the exercise in this study might have been more intense than in previous GE studies, which likely remained below ventilatory or lactate thresholds (11, 12). Positive affective responses normally resulted from the situation that exercise intensities below these thresholds, whereas intensities above them often lead to discomfort for most recreational exercisers (46). However, self-pacing can enhance tolerance to higher intensities better than imposed pacing. This self-regulation may have mitigated the negative effects of high-intensity exercise on their overall affect for some individuals (46). The results of both enjoyment of current exercise and intention for future exercise behavior confirmed this theory in both groups.

Previous research suggests that GE offers greater psychological benefits than exercising indoors or in artificial outdoor environments (10, 11, 15, 46). This finding implies that the additional affective benefits may be obtainable through a variety of GE environments. However, our study did not have a non-nature-based exercise environment for comparison. Since acute affective improvement of single exercise bouts are consistently reported, it is hard to distinguish between the contributions of the ‘greenness’ of the exercise environments and the exercise itself in this study. On the other hand, ‘non-green’ exercise may also provide similar affective benefits.

Functional outcomes over 24 weeks show that the Green outdoor group experienced larger improvements in HbA1c, BMI, and KOOS-QoL compared to the ISC group (P < 0.05). Improvements in KOOS-ADL and KOOS-Sport/Rec were also significantly greater in the OGC group​. Englund suggested that the modest benefits of exercise interventions for KOA could largely be attributed to the placebo effect, the disease’s natural progression, and statistical regression to the mean (47). Thus, our findings implied that placebo effects of exercise have less connection with environment. Some authors considered the use of exercise treatment in chronic pain conditions should be viewed as a form cognitive therapy, where the goal is to modulate the feeling of pain and thus patients’ thoughts and feelings about it rather than increasing muscle strength and endurance (48, 49).

A key strength of this study was its ecological validity. The results reflect improvements in psychological well-being observed in a real-world setting with individuals acting on their own, rather than through a designed and instructed intervention. This study also has several limitations. First, in the baseline condition, the submaximal test was conducted after participants completed a brief warm-up cycling session. For participants who exercise less frequently, even “extremely light” intensity exercise could influence the heart rate response of a subsequent bout, potentially biasing the estimated 50% HRR intensity levels used for the experimental conditions. To mitigate this potential bias, resting heart rate values were obtained earlier during the baseline session, and participants took a two-minute rest period immediately before the submaximal test. Second, a limitation of this study is the relatively short follow-up period of 24 weeks, which may not fully capture the long-term benefits of green exercise. While this duration is appropriate for assessing immediate and short-term changes in psychosocial and physiological outcomes, it does not provide insights into the sustainability of these effects. Future studies with extended follow-up periods are needed to evaluate whether the positive outcomes observed during the study persist over time and contribute to long-term improvements in both KOA and T2DM. Thirdly, a potential source of bias in our study is observational bias, particularly in relation to the psychosocial outcomes, such as self-esteem, perceived stress, and mood. Participants in the green exercise group may have experienced positive social and environmental factors—such as being outdoors and engaging in group exercise—that could have influenced their responses to these measures. Lastly, while baseline radiographic characteristics (including Kellgren-Lawrence grade and patellofemoral OA status) were documented, longitudinal imaging data were not systematically collected due to ethical and practical constraints. Future studies might prioritize serial radiographic assessments to evaluate structural progression alongside clinical outcomes, providing deeper insights into the relationship between exercise modalities and OA pathophysiology.

Conversely, participants in the indoor cycling group may have been less exposed to such external influences. This could result in differences in reported psychosocial outcomes that are not solely attributable to the exercise modality but may also reflect the impact of the environment in which the exercise took place. We acknowledge that these biases may have influenced the observed differences in psychosocial outcomes and recommend that future studies incorporate strategies to control for such factors, such as blinding or more objective measures of psychosocial well-being. Furthermore, another limitation of our study is the lack of control for environmental factors, such as air quality, temperature, and weather conditions, which may have influenced the outcomes observed in the OGC group. Future studies should consider controlling for environmental factors, either by monitoring these variables or by conducting exercises in more controlled outdoor settings, to better isolate the effects of green exercise from other external influences.

## Conclusion

Green exercise enhances self-esteem and mood while reducing stress, regardless of the type of green setting, and this is particularly beneficial for patients coping with chronic conditions like T2DM and OA.

## Data Availability

The raw data supporting the conclusions of this article will be made available by the authors, without undue reservation.
