# Supplementary material for: Psychosocial and physiological health outcomes of outdoor green exercise versus indoor exercise in knee osteoarthritis patients coexisting with type 2 diabetes mellitus: a randomized controlled trial
Source: Front Endocrinol (Lausanne). 2025 May 14;16:1560536. doi: 10.3389/fendo.2025.1560536 (PMC12116314; doi:10.3389/fendo.2025.1560536)
Supplement: Supplementary file 1 [file DataSheet1.doc]

**Supplemental Materials**

**Process of Posture Setting for Cycling**

1. Bike Fit Adjustment

Before focusing on posture, ensure your bike is properly fitted to your body dimensions.

Saddle Height:

- - Sit on the saddle and place your heel on the pedal.
  - Pedal backward until your leg is fully extended at the lowest pedal position.
  - Your leg should be straight, indicating the correct saddle height.

Saddle Position:

- - The saddle should be level and not tilted excessively up or down.
  - Ensure the saddle is not too far forward or backward. When the pedals are horizontal, the front of your kneecap should be directly above the pedal axle.

Handlebar Height:

- - For most cyclists, the handlebars should be approximately level with or slightly below the saddle height.
  - Adjust the handlebars based on your flexibility and comfort. Higher handlebars can be more comfortable for those with back or neck issues.

2. Posture on the Bike

Hand Position:

- - Hold the handlebars with a relaxed grip, keeping your wrists straight and in line with your forearms.
  - Change hand positions regularly to avoid numbness and fatigue.

Shoulder and Arm Position:

- - Keep your shoulders relaxed and away from your ears.
  - Your elbows should be slightly bent, not locked, to absorb shocks and maintain control.

Back Position:

- - Maintain a neutral spine position. Your back should be straight, not hunched, with a slight natural curve.
  - Engage your core muscles to support your lower back and maintain stability.

Knee Alignment:

- - Your knees should be aligned with your feet, not splaying out or in.
  - When viewed from the front, your knees should track straight up and down, avoiding lateral movement.

Foot Position:

- - Place the ball of your foot on the pedal, with your foot parallel to the ground at the midpoint of the pedal stroke.
  - Use cycling shoes and clip-in pedals if possible to ensure a secure and efficient pedal stroke.

3. Pedaling Technique

Smooth Pedal Stroke:

- - Aim for a smooth, circular pedal stroke rather than pushing down hard on each pedal.
  - Visualize scraping mud off the bottom of your shoe as you bring the pedal up, emphasizing an even force application throughout the rotation.

Cadence:

- - Maintain a steady cadence, typically between 60-90 revolutions per minute (RPM) for most recreational cyclists.
  - Avoid mashing the pedals at a low cadence to prevent excessive strain on your knees.

4. Body Awareness and Adjustments

Regular Checks:

- - Periodically check your posture during your ride. Make small adjustments to your position to stay comfortable and avoid developing bad habits.
  - Use mirrors or ask a cycling partner to observe and provide feedback on your posture.

Flexibility and Strength:

- - Incorporate stretching and strength training into your routine to improve flexibility and core strength, which will support better posture on the bike.
  - Focus on exercises that target the hamstrings, quadriceps, glutes, lower back, and core muscles.

**Exercise Programs**

Indoor Stationary Cycling Program for Knee Osteoarthritis Patients Coexisting with Type 2 Diabetes Mellitus

Week 1-2: Initial Phase

Objective: Adaptation to exercise, focus on low intensity.

1. Warm-up: 5 minutes of slow pedaling at a comfortable pace (RPE 2-3).
2. Main Session:
   - 20 minutes of continuous cycling at low intensity (50-60% of Max HR or RPE 3-4).
   - Cadence: 60-70 RPM.
3. Cool-down: 5 minutes of slow pedaling and gentle stretching focusing on the lower limbs.

Week 3-4: Progression Phase

Objective: Gradual increase in duration and intensity.

1. Warm-up: 5 minutes of slow pedaling at a comfortable pace (RPE 2-3).
2. Main Session:
   - 25 minutes of continuous cycling at moderate intensity (60-70% of Max HR or RPE 4-5).
   - Cadence: 70-80 RPM.
3. Cool-down: 5 minutes of slow pedaling and gentle stretching focusing on the lower limbs.

After week 5: Advanced Phase

Objective: Enhance cardiovascular fitness and muscle endurance.

1. Warm-up: 5 minutes of slow pedaling at a comfortable pace (RPE 2-3).
2. Main Session:
   - 30 minutes of continuous cycling at moderate intensity (65-75% of Max HR or RPE 4-6).
   - Include 2-3 intervals of 2 minutes at higher intensity (75-85% of Max HR or RPE 6-7) with 3 minutes of recovery between intervals.
   - Cadence: 70-80 RPM.
3. Cool-down: 5 minutes of slow pedaling and gentle stretching focusing on the lower limbs.

Target Heart Rate Calculation

- Maximum Heart Rate (Max HR) = 220 - age.
- For moderate intensity: Target HR = 60-70% of Max HR.

Outdoor Cycling Program for Knee Osteoarthritis Patients Coexisting with Type 2 Diabetes Mellitus

Week 1-2: Initial Phase

Objective: Adaptation to outdoor conditions, focus on low intensity.

1. Warm-up: 5 minutes of slow cycling on flat terrain (RPE 2-3).
2. Main Session:
   - 20 minutes of continuous cycling on flat terrain at low intensity (50-60% of Max HR or RPE 3-4).
   - Maintain a steady pace.
3. Cool-down: 5 minutes of slow cycling and gentle stretching focusing on the lower limbs.

Week 3-4: Progression Phase

Objective: Gradual increase in duration and introduction to slight inclines.

1. Warm-up: 5 minutes of slow cycling on flat terrain (RPE 2-3).
2. Main Session:
   - 25 minutes of continuous cycling including gentle inclines at moderate intensity (60-70% of Max HR or RPE 4-5).
   - Focus on smooth, controlled pedaling.
3. Cool-down: 5 minutes of slow cycling and gentle stretching focusing on the lower limbs.

After week 5: Advanced Phase

Objective: Enhance cardiovascular fitness and muscle endurance, handle varying terrain.

1. Warm-up: 5 minutes of slow cycling on flat terrain (RPE 2-3).
2. Main Session:
   - 30 minutes of continuous cycling including moderate inclines at moderate intensity (65-75% of Max HR or RPE 4-6).
   - Include 2-3 intervals of 2 minutes on steeper inclines at higher intensity (75-85% of Max HR or RPE 6-7) with 3 minutes of recovery on flat terrain between intervals.
3. Cool-down: 5 minutes of slow cycling and gentle stretching focusing on the lower limbs.

Target Heart Rate Calculation

- Maximum Heart Rate (Max HR) = 220 - age.
- For moderate intensity: Target HR = 60-70% of Max HR.

1. Intensity Monitoring: Both programs use the Rate of Perceived Exertion (RPE) and Heart Rate (HR) to ensure exercise intensity is appropriate for cardiovascular and joint health.
2. Adaptation Phases: The gradual increase in duration and intensity helps prevent overuse injuries and accommodate the limitations imposed by knee osteoarthritis.
3. Terrain and Cadence: For outdoor cycling, terrain variation is introduced progressively to manage joint stress and improve muscle strength.
4. Diabetes Management: Regular, moderate-intensity aerobic exercise is recommended for managing blood glucose levels, which is integrated into the program.

References:

1. Kolasinski SL, Neogi T, Hochberg MC, Oatis C, Guyatt G, Block J, et al. 2019 American College of Rheumatology/Arthritis Foundation Guideline for the Management of Osteoarthritis of the Hand, Hip, and Knee. Arthritis Care Res (Hoboken). 2020;72(2):149-162.
2. Balducci S, Sacchetti M, Haxhi J, Orlando G, D'Errico V, Fallucca S, Menini S, Pugliese G. Physical exercise as therapy for type 2 diabetes mellitus. Diabetes Metab Res Rev. 2014;30 Suppl 1:13-23.
